# Neuromedin B Restores Erectile Function by Protecting the Cavernous Body and the Nitrergic Nerves from Injury in a Diabetic Rat Model

**DOI:** 10.1371/journal.pone.0133874

**Published:** 2015-07-24

**Authors:** Hiroaki Nishimatsu, Etsu Suzuki, Yasuho Saito, Aya Niimi, Akira Nomiya, Daisuke Yamada, Yukio Homma

**Affiliations:** 1 The Department of Urology, Faculty of Medicine, University of Tokyo, 7-3-1 Hongo, Bunkyo-ku, Tokyo, 113–8655, Japan; 2 Institute of Medical Science, St. Marianna University School of Medicine, 2-16-1 Sugao, Miyamae-ku, Kawasaki, 216–8512, Japan; Max-Delbrück Center for Molecular Medicine (MDC), GERMANY

## Abstract

Erectile dysfunction (ED) is a major health problem worldwide and affects approximately 75% of diabetic patients, likely due to severely damaged cavernous body. While screening for cytokines produced by adipose tissue-derived stem cells, we detected neuromedin B (NMB). To explore a potential treatment option for ED, we examined whether NMB was capable of restoring erectile function. We also examined the potential mechanism by which NMB could restore erectile function. Male Wistar rats were injected with streptozotocin (STZ) to induce diabetes. An adenovirus expressing NMB (AdNMB) was injected into the penis 6 weeks after STZ administration. Four weeks after the injection of AdNMB, erectile function, penile histology, and protein expression were analyzed. As assessed by the measurement of intracavernous pressure, AdNMB injection significantly restored erectile function compared with the injection of an adenovirus expressing green fluorescent protein. This restoration was associated with conservation of the cavernous body structure and neural nitric oxide synthase (nNOS)-expressing nerves, together with recovery of α-smooth muscle actin, vascular endothelial-cadherin, and nNOS expression. Furthermore, NMB significantly stimulated the survival of SH-SY5Y cells derived from human neuroblastoma tissue with characteristics similar to neurons. Collectively, these results suggested that NMB restored erectile function via protection of the cavernous body from injury and stimulation of the survival of the associated nerves. NMB may be useful to treat ED patients with a severely damaged cavernous body.

## Introduction

Erectile dysfunction (ED) is a major health problem worldwide. It is also a common complication of diabetes in men, estimated to affect approximately 75% of these patients [1]. Although selective phosphodiesterase type 5 inhibitors (PDE-5Is) are considered to be a first-line therapy for ED, PDE-5Is are less effective in diabetic patients [2, 3], likely due to the severely damaged cavernous body in these patients. To effectively treat ED in diabetic patients, it is therefore necessary to explore novel methods for the regeneration of the cavernous body, both morphologically and functionally.

Many studies using gene therapy and cell therapy have demonstrated that erectile function could be restored, at least in animal models [4–15]. Among a variety of adult stem cells, bone marrow-derived mesenchymal stem cells (BMMSCs) and adipose tissue-derived stem cells (ASCs), which are derived from subcutaneous adipose tissue, have been widely used to treat ED in animal models [8, 10–15]. Although both BMMSCs and ASCs are effective for the treatment of ED, the mechanisms by which they restore erectile function remain controversial. In some studies, BMMSCs and ASCs were engrafted in the penile tissue and expressed markers for vascular endothelial cells (VECs), vascular smooth muscle cells, and/or nerves [8, 12, 13, 15]. However, engraftment was barely detected and paracrine factors secreted from these stem cells appeared to play a major role in the restoration of erectile function in other studies [10, 11, 14]. We have also shown that the administration of ASCs protected the cavernous body from injury and restored erectile function in diabetic rats [16]. We have demonstrated that ASCs prevented the destruction of the cavernous body by secreting cytokines that stimulated angiogenesis, as opposed to being integrated in the cavernous body itself [16]. Among the various cytokines produced by ASCs, we identified adrenomedullin (AM) and angiopoietin-1 (Ang-1) as molecules that effectively protected the cavernous body from injury [17].

During additional screening for cytokines produced by ASCs, we recently detected neuromedin B (NMB), which is a member of a family of mammalian bombesin-like peptides [18]. NMB has demonstrated various activities including stimulation of the contraction of gastrointestinal/urogenital smooth muscles [19–21], inhibition of thyrotropin release from the pituitary gland [22, 23], induction of satiety [24, 25] and mediation of stress and fear responses [26, 27]. Additionally, expression of the receptor for NMB has been detected in VECs [28, 29]. Furthermore, NMB can stimulate angiogenesis in VECs [29]. Collectively, these results suggested that NMB had the potential to promote angiogenesis in the cavernous body and regenerate its structure. In this study, we examined whether NMB had the ability to restore erectile function and maintain the structure and function of the cavernous body.

## Materials and Methods

### Reagents

Human NMB and human nerve growth factor (NGF) were obtained from the Peptide Institute (Catalogue NO. 4152-v, Osaka, Japan) and Wako Pure Chemical Industries (Catalogue NO. 141–07601, Tokyo, Japan), respectively.

### Cell culture

ASCs were isolated from male Wistar rats as previously described [30]. ASCs were then cultured on fibronectin-coated dishes in endothelial growth medium-2MV (EGM; Lonza Walkersville, Inc, Walkersville, MD). The EGM consisted of endothelial basal medium-2 (Lonza Walkersville, Inc., Walkersville, MD) containing 5% fetal bovine serum (FBS) plus growth factors, such as epidermal growth factor, hydrocortisone, vascular endothelial growth factor-A (VEGF-A), basic fibroblast growth factor, and insulin-like growth factor-1 (IGF-1). ASCs were also cultured on fibronectin-coated dishes in endothelial basal medium-2 containing 5% FBS (EBM) as a negative control. SH-SY5Y cells derived from human neuroblastoma tissue were purchased from the American Type Culture Collection (ATCC, Manassas, VA), and were cultured in a 1:1 mixture of Dulbecco’s modified Eagle medium (DMEM) and F12 medium (DMEM-F12 (1:1)) containing 10% FBS. HEK293 cells (ATCC) were cultured in DMEM containing 10% FBS.

### Construction of an adenovirus that expressed rat NMB

A replication-defective adenovirus that expressed rat NMB was constructed according to the previously described method using an AdMax kit (Microbix Biosystems Inc., Ontario, Canada) [31]. The coding region of the rat NMB was amplified by polymerase chain reaction (PCR) and subcloned into the pDC516 vector. The primer sequences used for PCR were as follows:

RatNMBsense primer: 5’-ATGACCCGGCAAGCAGGGAGCA-3’

RatNMBantisense primer: 5’-TCACTTCTGCAGCAGCCTCCTGT-3’

After determining the DNA sequence, the expression plasmid pDC516 that contained the rat NMB sequence was cotransfected into HEK293 cells with pBHGfrtdelE13FLP to construct an adenovirus that expressed rat NMB (AdNMB). A recombinant adenovirus that expressed green fluorescent protein (AdGFP) was obtained from Quantum Biotechnologies (Montreal, Canada).

### Animal experiments

All procedures using experimental animals were approved by the Institutional Committee for Animal Research of the Tokyo University. Streptozotocin (STZ; 50 mg/kg body weight; Sigma-Aldrich, Tokyo, Japan) was dissolved in citrate buffer (pH 4.5) and injected into the tail vein of male Wistar rats (6 weeks of age; Charles River, Wilmington, MA). Blood glucose levels were measured 4 weeks later to confirm that these mice became diabetic. Either AdNMB or AdGFP was injected into the penis (18 rats per group) 6 weeks after STZ injection. Four weeks after adenovirus injection, rats (16 weeks of age) were subjected to an intracavernous pressure (ICP) measurement (5 rats per group). Penises were also harvested for histochemical analysis (4 rats per group) and western blot analysis (4 rats per group) at this time point. The penises isolated from the remaining 5 rats per group were used for real-time PCR analysis. Age-matched Wistar rats (positive controls, a total of 13 rats) were also used for ICP measurement (5 rats), histochemical analysis (4 rats), and western blot analysis (4 rats). ICP measurement was performed as previously described [32]. Rats were anesthetized with ketamine (100 mg/kg body weight) by intraperitoneal injection. The left carotid artery was cannulated with a PE-50 polyethylene tube to allow for continuous monitoring of mean arterial pressure (MAP). The penis was denuded of skin and cannulated with a 23-gauge needle connected to a pressure transducer in order to monitor ICP continuously. The pelvic and cavernous nerves were isolated, and the right cavernous nerve was hooked with a stainless steel bipolar electrode (TN-98119A, Unique Medical Co., Tokyo, Japan) connected to a nerve stimulator (Nihon Kohden Co., Tokyo, Japan). Electrical stimulation of the cavernous nerve was performed using a square wave stimulator under the following parameters: stimulation duration, 10 s; voltage, 5.0 V; pulse duration, 2 ms; and stimulation frequency, 20 Hz. The area under the curve (AUC) of the ICP traces, as well as the ratio of peak ICP to MAP (ICP/MAP), were used to evaluate erectile function. The cavernous nerve was stimulated at least 3 times in each rat, and the results were averaged to calculate the ICP/MAP.

### RNA extraction and real time PCR

Penis tissues were homogenized in TRIZOL Reagent (Life Technologies, Tokyo, Japan) to extract total RNA. Reverse transcription of total RNA was performed using a ReverTra Ace qPCR RT Kit (TOYOBO, Osaka, Japan). The expression of rat NMB and glyceraldehyde 3-phosphate dehydrogenase (GAPDH) was examined by real-time PCR using a SYBR Green dye (Thunderbird SYBR qPCR Mix, TOYOBO, Japan). Primers used were as follows:

RatNMBsense, 5’- TGTTCGTTTCCGGCATCAC -3’
RatNMBantisense, 5’- TGCACTCGAATCTTGCTTGCT -3’
RatGAPDHsense, 5’-GTATGACTCTACCCACGGCAAGT-3’
RatGAPDHantisense, 5’-TTCCCGTTGATGACCAGCTT-3’.

Real-time PCR was performed using an ABI PRISM 7000 sequence detection system (Applied Biosystems, Foster City, CA).

### Protein extraction and western blot analysis

To extract proteins, penis tissues were homogenized in a cell lysis buffer (50 mM Tris-HCl (pH 8.0), 150 mM NaCl, 1% NP-40) containing 2 μg/mL aprotinin, 2 μg/mL leupeptin and 1 mM phenylmethylsulfonyl fluoride. The western blot analysis was performed as previously described [33]. The dilutions of the antibodies used in this study were as follows: anti-vascular endothelial cadherin (VE-Cad) antibody: 1:200 (sc-9989, Santa-Cruz); anti-α-smooth muscle actin (SMA) antibody: 1:200 (sc-130617, Santa-Cruz); anti-neural nitric oxide synthase (nNOS) antibody: 1:250 (LS-C37730, LifeSpan BioSciences); anti-phospho-endothelial nitric oxide synthase (eNOS) antibody: 1:500 (9571, Cell Signaling); anti-eNOS antibody: 1:500 (9572, Cell Signaling); anti-β-actin antibody: 1:300 (sc-47778, Santa-Cruz).

### Histochemistry

Penis tissues were fixed by perfusion with 4% paraformaldehyde and then embedded in paraffin. The specimens were sliced at a thickness of 5 μm, deparaffinized, rehydrated, and subjected to histochemical analysis. Elastica van Gieson staining was performed according to the standard method. In brief, the specimens were stained with resorcin fuchsin staining solution (Muto Pure Chemicals, CO., LTD., Tokyo, Japan), hematoxylin staining solution, and van Gieson staining solution (Muto Pure Chemicals, CO., LTD., Tokyo, Japan). For immunohistochemistry, sections were incubated with a primary antibody reactive to VE-Cad (1:400; LS-B2138; LifeSpan BioSciences), SMA (1:200; ab18147; Abcam) or nNOS (1:400; ab76067; Abcam). Sections were then incubated with biotinylated secondary antibody prior to horseradish peroxidase-labeled streptavidin according to the manufacturer's instructions (DAKO, Cambridgeshire, UK).

### Enzyme-linked immunosorbent assay (ELISA)

The rat NMB accumulated in culture medium was measured using an ELISA kit (CEA803Ra, USCN Life Science, Hubei, China) according to the manufacturer's protocol.

### Induction of neuronal differentiation

SH-SY5Y cells were cultured in DMEM-F12 (1:1) containing 1% FBS in the presence or absence of NMB (10^−8^ M or 10^−7^ M) or NGF (25 ng/mL). Neurite outgrowth was measured using ImageJ software (National Institutes of Health, Bethesda, MD).

### Cell survival assay

SH-SY5Y cells were plated in 96 well plates and cultured in DMEM-F12 (1:1) containing 10% FBS until they reached approximately 90% confluence. After washing with phosphate-buffered saline (PBS), the medium was changed to serum-free (without FBS) DMEM-F12 (1:1) plus NMB (10^−8^ M or 10^−7^ M) or NGF (25 ng/mL). Survival of the cells was assayed using the CellTiter 96 AQueous One Solution Cell Proliferation Assay kit (Promega, Tokyo, Japan) according to the manufacturer's protocol. Briefly, medium was replaced with 100 μL of phenol red-free DMEM, and 20 μL of CellTiter 96 AQueous One Solution reagent was added to each well. After incubation at 37°C for 1 hr, optical density was measured using a microplate reader at 450 nm.

### Statistical analysis

Values are expressed as the mean ± the standard error of the mean. Statistical analyses were performed using analysis of variance followed by the Student–Newman–Keuls test. P values<0.05 were considered to be statistically significant.

## Results

### ASCs produced NMB, particularly when cultured in EGM

**Fig 1:**
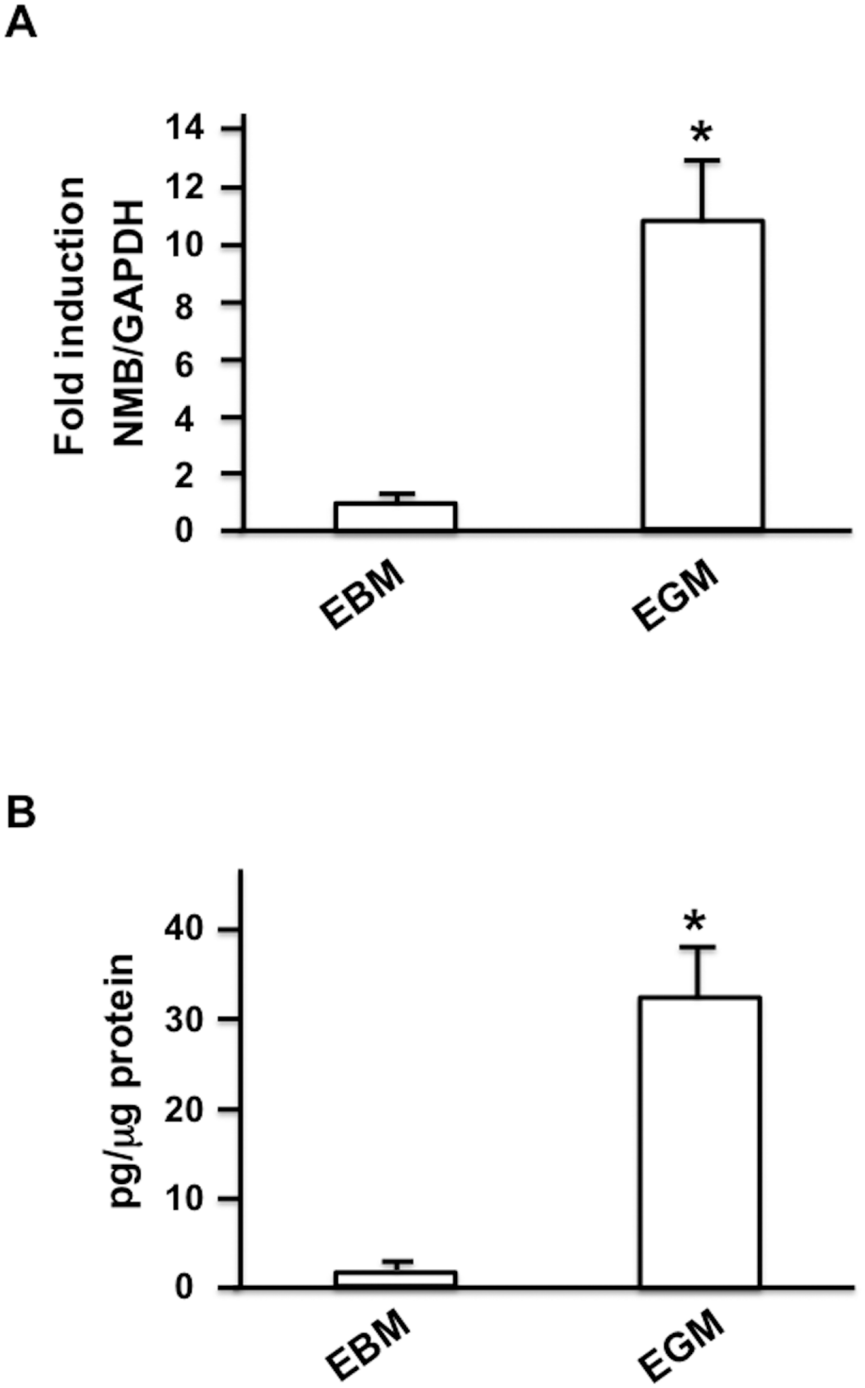
Expression of NMB in ASCs. (A) Expression of NMB mRNA. Rat ASCs were cultured in EBM and EGM for 7 days. Total RNA was extracted from ASCs and real time PCR analysis was performed in order to examine the expression of NMB (n = 6 per group). *: P<0.01 vs. EBM culture. (B) Expression of NMB protein by ASCs. After culturing ASCs in EBM and EGM for 7 days, the cells were washed with PBS, and the medium was changed to serum-free DMEM. After 2 h, the medium was collected and used for ELISA (n = 6 per group). *: P<0.01 vs. EBM culture.

**Fig 2:**
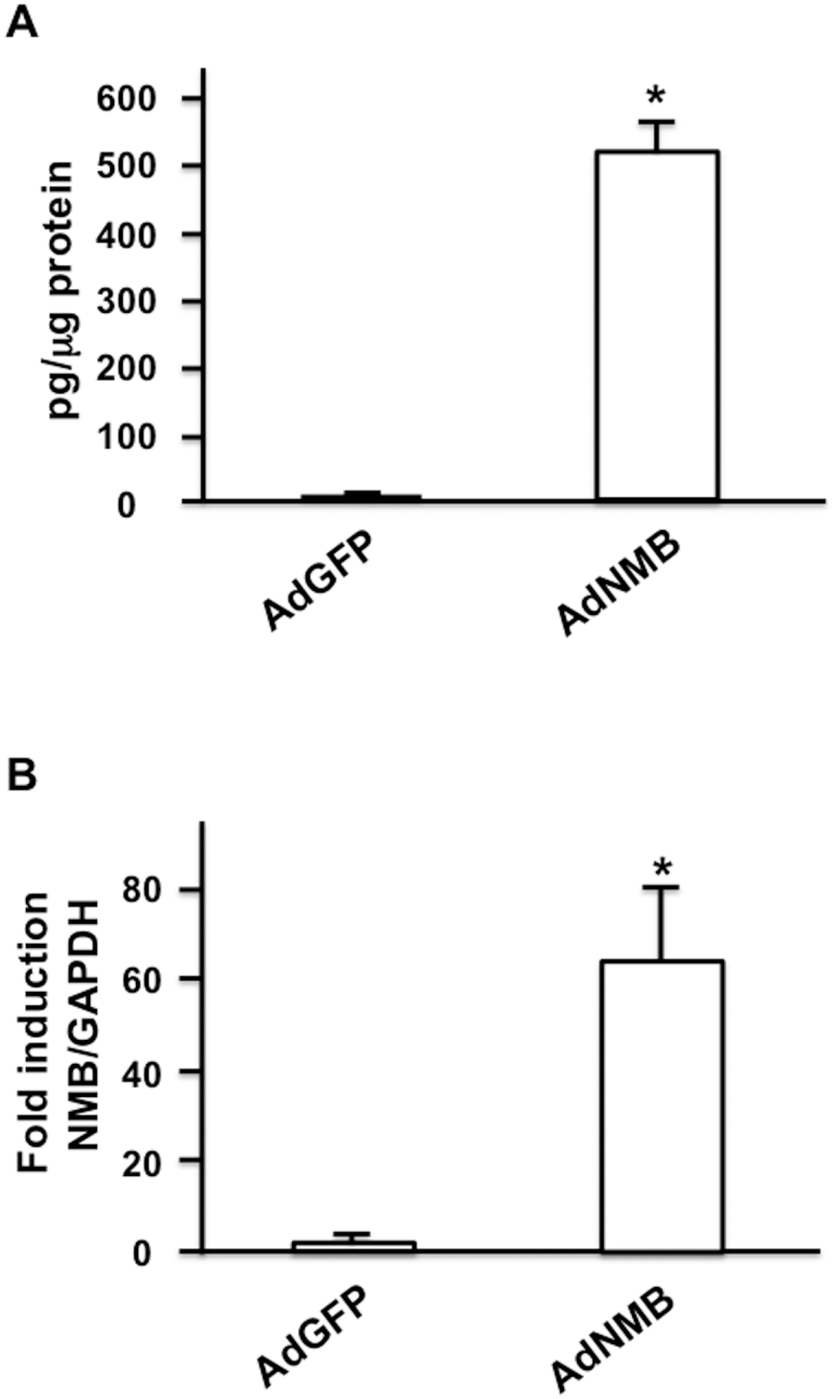
Expression of NMB by AdNMB. (A) Expression of NMB protein by AdNMB. HEK293 cells plated on type I collagen-coated dishes, were infected with AdNMB. After 24 h, the medium was replaced with serum-free DMEM, and the medium was collected 2 hours later for ELISA (n = 6 per group). *: P<0.001 vs. AdGFP infection. (B) Expression of NMB mRNA in the penis after AdNMB injection. AdGFP and AdNMB were injected into the penis and total RNA was extracted 7 days after injection. Real time PCR analysis was performed in order to examine the expression of NMB mRNA in the penis (n = 5 per group). *: P<0.01 vs. AdGFP infection.

We first examined the production of NMB in ASCs. When cultured in EGM, ASCs expressed significantly higher amounts of NMB mRNA than when cultured in EBM, as assessed by real time PCR analysis (Fig 1A). The expression of NMB protein was also measured by ELISA. ASCs produced significantly higher amounts of NMB protein when cultured in EGM than when cultured in EBM (Fig 1B). We next examined the expression of NMB protein using AdNMB. HEK293 cells were infected with AdNMB, and NMB secreted into the culture medium was measured by ELISA. HEK293 cells infected with AdNMB produced significantly greater amounts of NMB protein than those infected with AdGFP (Fig 2A), suggesting that the AdNMB used in this study produced NMB. AdNMB was also injected into the rat penis, and real time PCR analysis was performed to examine the expression of NMB mRNA in the penis 7 days later. The penises injected with AdNMB expressed significantly higher amounts of NMB mRNA than those injected with AdGFP (Fig 2B), suggesting that the AdNMB injected into the penises remained there and expressed NMB.

### NMB restored erectile function in diabetic rats

**Fig 3:**
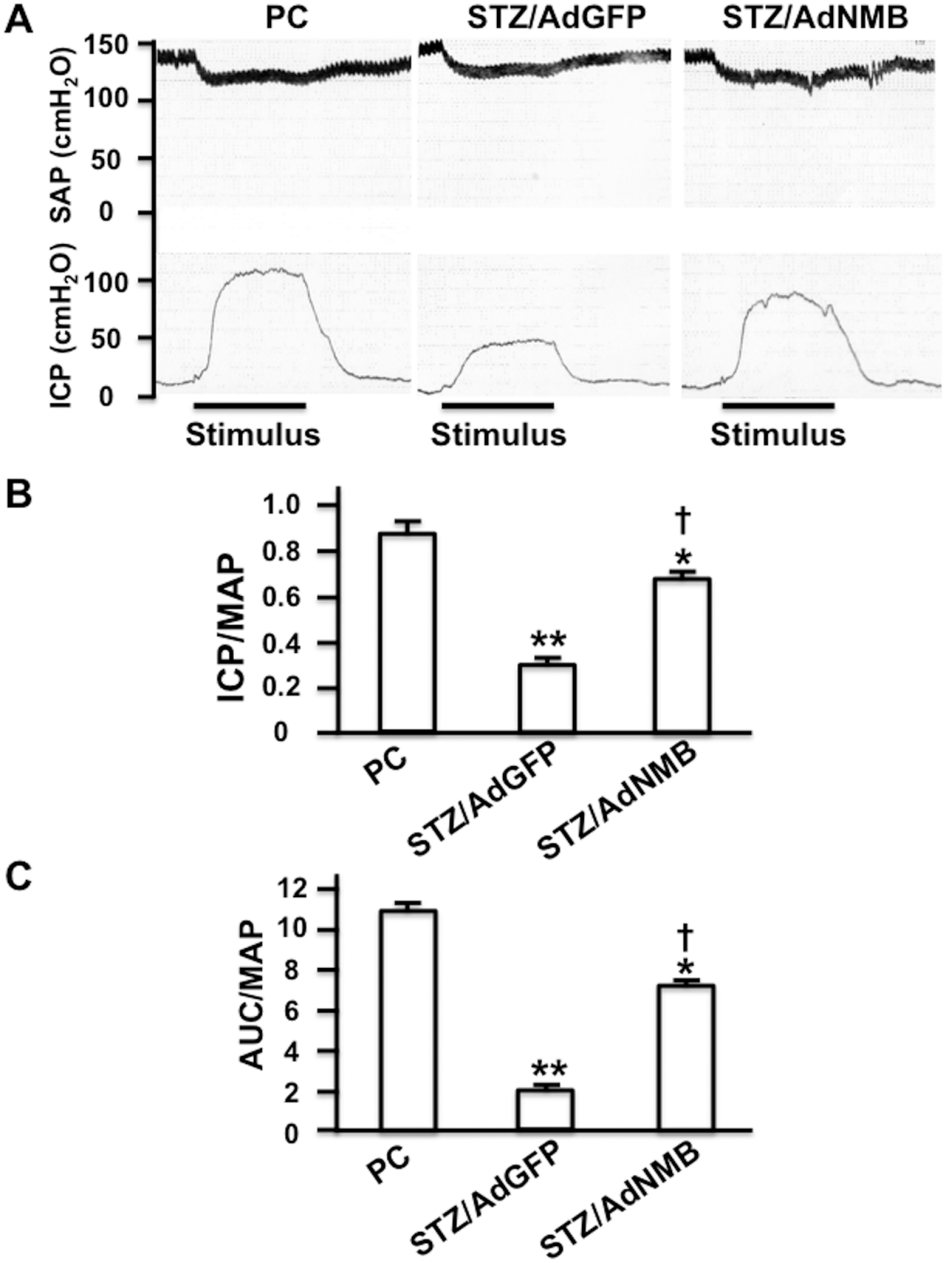
Overexpression of NMB restores erectile function. AdGFP (STZ/AdGFP) and AdNMB (STZ/AdNMB) were injected into the penises of STZ-induced diabetic rats, and ICP was measured 4 weeks later. Age-matched non-diabetic control rats were also used as positive controls (PC). (A) Representative traces of ICP and systemic arterial pressure (SAP). The stimulus interval (10 s) is indicated by a solid bar. (B) Histograms comparing the ICP/MAP among the groups (n = 5 per group). * and **: P<0.01 and P<0.001, respectively vs. PC. †: P<0.001 vs. AdGFP-injection. (C) Histograms comparing the AUC/MAP (n = 5 per group). * and **: P<0.01 and P<0.001, respectively vs. PC. †: P<0.001 vs. AdGFP-injection.

The ICP was measured in order to analyze erectile function. We used STZ-induced diabetic rats with AdGFP injected into the penis as a negative control since we have previously confirmed that the AdGFP injection does not affect erectile function in diabetic rats [16]. The ICP/MAP was significantly decreased in the AdGFP-injected group as compared with the age-matched positive control group. AdNMB injection significantly restored the ICP/MAP as compared with AdGFP-injection, although not to the same level observed in the positive control group (Fig 3B). We also analyzed the ratio of the AUC to the MAP (AUC/MAP), which was also significantly decreased in the AdGFP-injected group as compared with the positive control group. The AUC/MAP was restored in the AdNMB-injected group as compared with the AdGFP-injected group, although the AUC/MAP in the AdNMB-injected group did not return to the same level observed in the positive control group (Fig 3C).

### Histology of the penis

**Fig 4:**
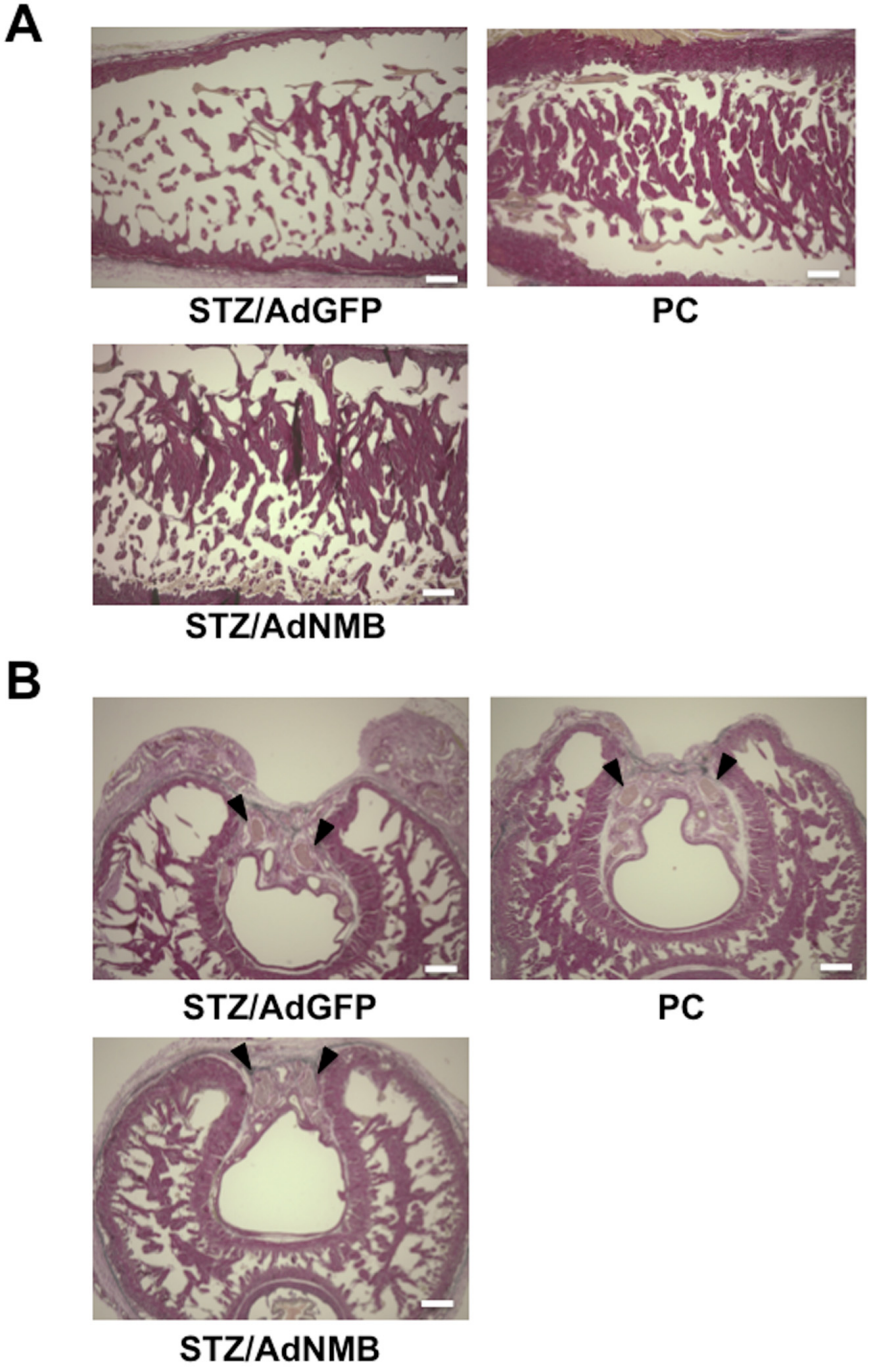
Elastica van Gieson staining of the cavernous body isolated from age-matched positive control rats and STZ-induced diabetic rats injected with AdGFP or AdNMB. AdGFP (STZ/AdGFP) and AdNMB (STZ/AdNMB) were injected into the penises of STZ-induced diabetic rats and the penises were harvested for Elastica van Gieson staining 4 weeks later. Age-matched non-diabetic rats were used as positive controls (PC). (A) The histology of the root portion of penis (longitudinal section) is shown. Bars are 300 μm. (B) The histology of the cross section of penis is shown. Arrowheads indicate the dorsal penile nerves. Bars are 300 μm.

**Fig 5:**
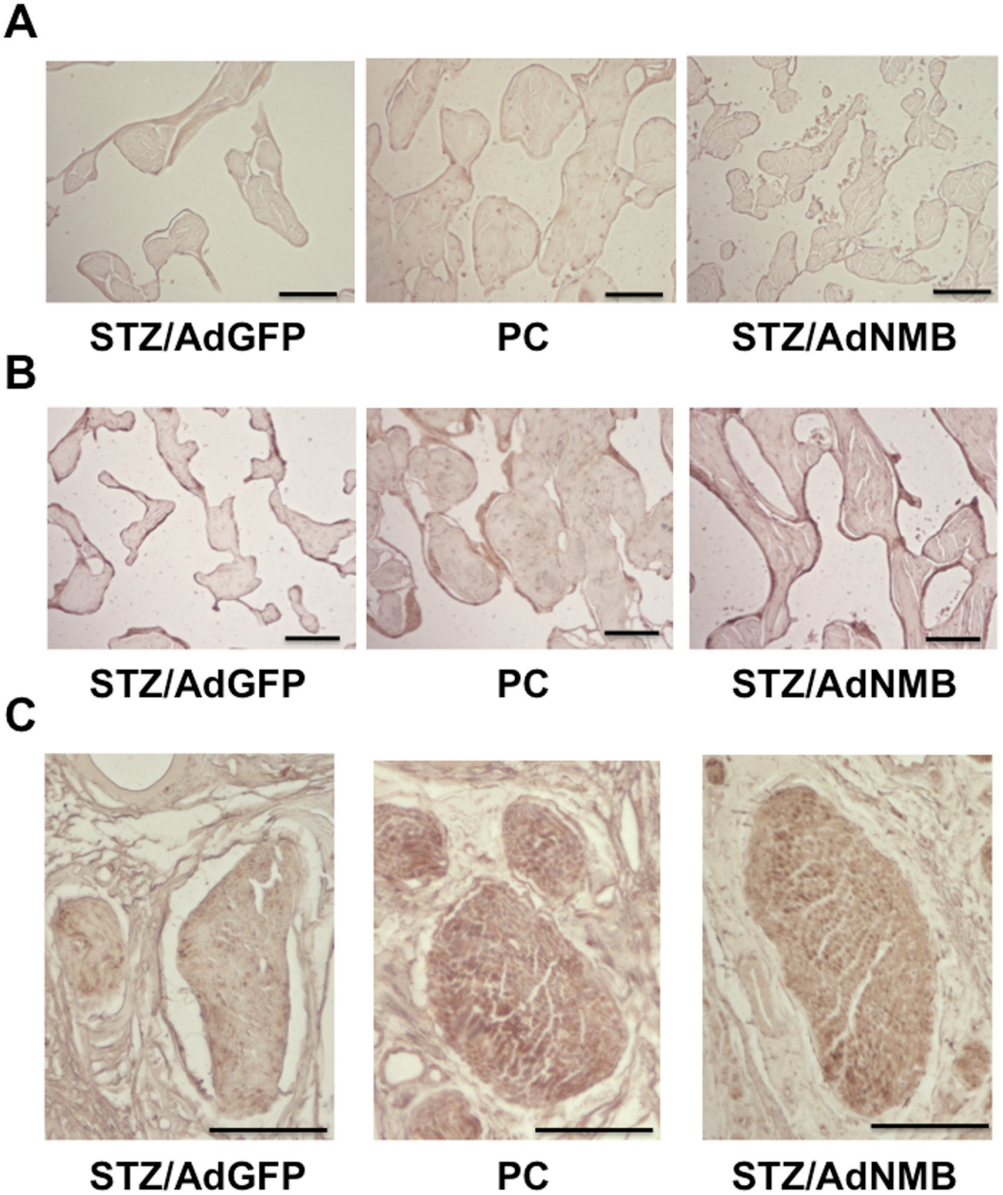
Immunohistochemical analyses of the penis. Experiments were performed in the same manner as described in Fig 4 legend. (A) Immunostaining of VE-Cad in the cavernous body. The histology of the root portion of penis (longitudinal section) is shown. Scale bars are 100 μmeter. (B) Immunostaining of SMA in the cavernous body. The histology of the root portion of penis (longitudinal section) is shown. Scale bars are 100 μmeter. (C) Immunostaining of nNOS in the dorsal penile nerve. The histology of the cross section of penis is shown. Scale bars are 100 μmeter.

Elastica van Gieson staining was performed to observe morphological changes. Trabeculae of the cavernous body decreased in size in STZ-induced diabetic rats injected with AdGFP compared with those in age-matched positive control rats (Fig 4A). When AdNMB was injected into the penis, the size of trabeculae increased compared with that in AdGFP-injected diabetic rats and remained similar to that in positive control rats. The morphology of dorsal penile nerve did not change remarkably among the groups (Fig 4B). An immunohistochemical analysis was subsequently performed. VE-Cad immunostaining was observed on the surface of trabeculae of the cavernous body even in STZ-induced diabetic rats injected with AdGFP (Fig 5A). SMA was also immunostained on the surface of the trabeculae in STZ-induced diabetic rats injected with AdGFP (Fig 5B). However, the SMA-positive area looked thinner than that observed in the age-matched positive control rats. The SMA-positive area became thicker in the AdNMB-injected diabetic rats than in the AdGFP-injected diabetic rats. Immunostaining for nNOS in the dorsal penile nerve was also analyzed, as it has been reported that nitric oxide (NO) released from nitrergic nerves [34], which express nNOS and release NO as a transmitter, play pivotal roles in the initiation of erection [35]. The level of immunostaining for nNOS was remarkably decreased in the AdGFP-injected diabetic rats as compared with the age-matched positive control rats (Fig 5C). AdNMB injection restored the nNOS immunostaining as compared with the AdGFP-injected diabetic rats.

### Protein expression

**Fig 6:**
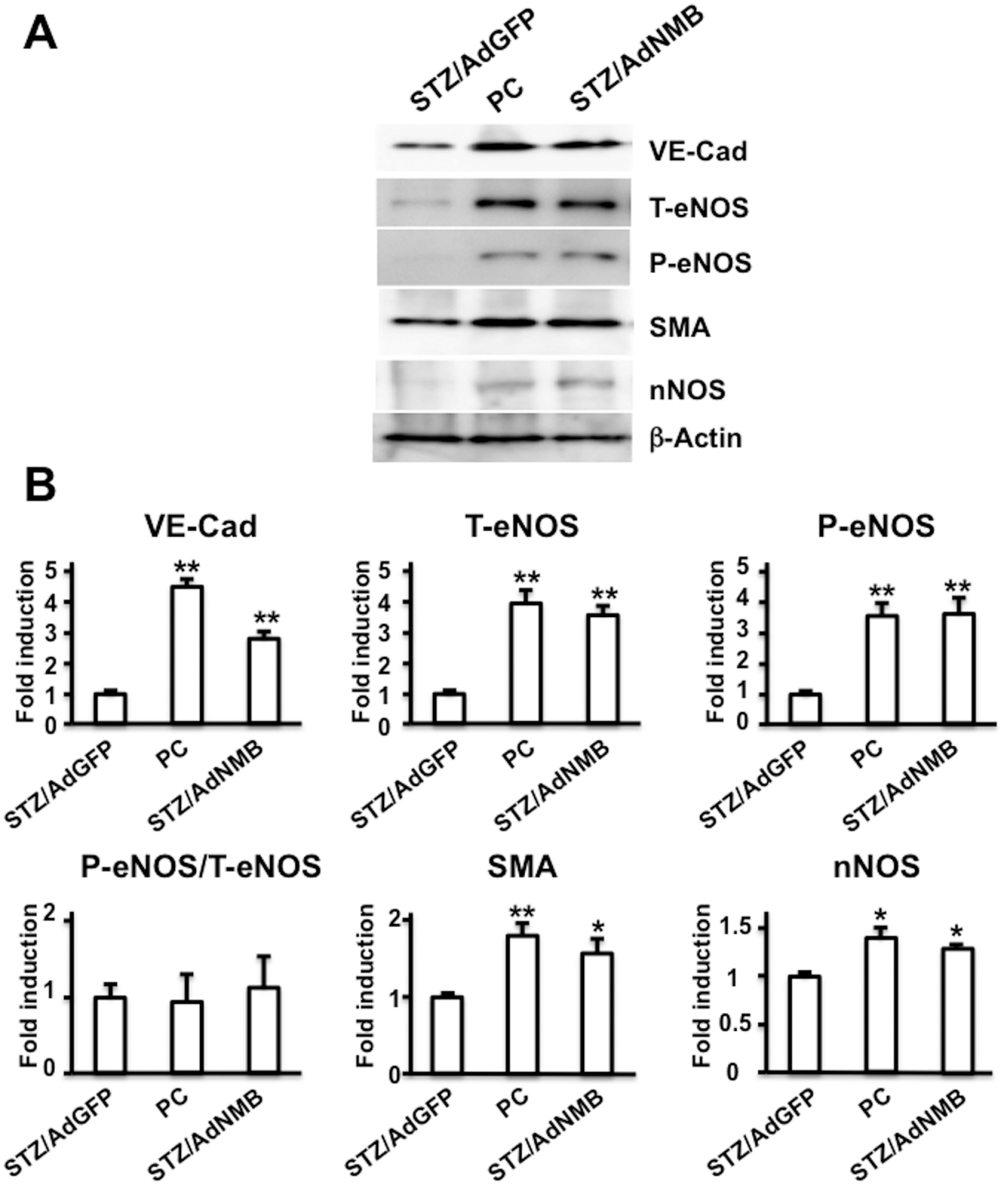
Expression of VE-Cad, eNOS, SMA, and nNOS proteins in the penis. AdGFP (STZ/AdGFP) and AdNMB (STZ/AdNMB) were injected into the penises of STZ-induced diabetic rats and the penises were harvested for protein extraction 4 weeks later. Age-matched non-diabetic rats were used as positive controls (PC). Western blot analysis was performed to detect the expression of VE-Cad, total eNOS (T-eNOS), phospho-eNOS (P-eNOS), SMA, and nNOS. (A) The representative images of the expression of VE-Cad, total eNOS, phospho-eNOS, SMA, and nNOS are shown. (B) Bar graphs comparing the expression levels of VE-Cad, total eNOS, phospho-eNOS, SMA, and nNOS among the groups (n = 4 per group). * and **: P<0.05 and P<0.01, respectively vs. AdGFP-injected diabetic rats.

To quantify the amount of VE-Cad, SMA, and nNOS expressed in the penis, western blot analysis was performed (Fig 6). The expression of VE-Cad, SMA, and nNOS decreased significantly in the AdGFP-injected diabetic rats as compared with the age-matched positive control rats. The expression levels of VE-Cad, SMA, and nNOS were restored significantly in the AdNMB-injected diabetic rats as compared with the AdGFP-injected diabetic rats. The expression of eNOS and phospho-eNOS, which is phosphorylated at Ser1177 and is catalytically active, was also examined. The expression of both total eNOS and phospho-eNOS significantly decreased in AdGFP-injected diabetic rats compared with that in positive control rats. These expressions were restored significantly in AdNMB-injected diabetic rats compared with those in AdGFP-injected diabetic rats. The ratio of phospho-eNOS to total eNOS expression was not significantly different among the groups, due to parallel changes in eNOS expression and phosphorylation.

### Effects of NMB on neuron differentiation and survival

**Fig 7:**
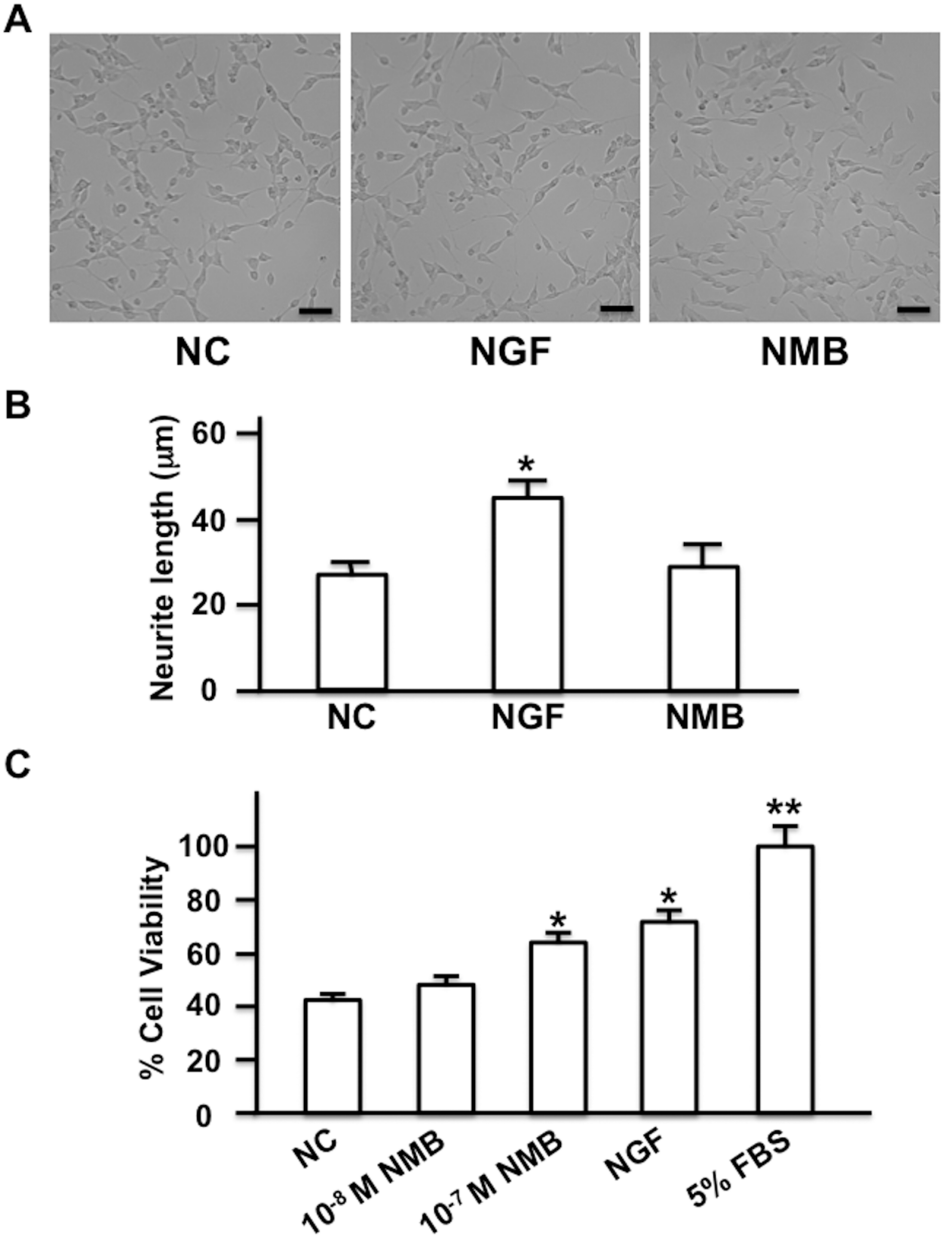
Effects of NMB on the differentiation and survival of SH-SY5Y cells. (A) Effect of NMB on the differentiation of SH-SY5Y cells. SH-SY5Y cells were cultured in DMEM-F12 (1:1) containing 1% FBS in the presence of NMB (10^−7^ M) or NGF (25 ng/mL) for 3 days. SH-SY5Y cells cultured in DMEM-F12 (1:1) containing 1% FBS were used as negative controls (NC). Representative photographs of the neurites are shown. Scale bars are 50 μmeter. (B) Bar graphs comparing the length of neurites. One hundred of neurites were randomly selected in each group and the length of the neurites was measured. *: P<0.01 vs. NC. (C) Effect of NMB on the survival of SH-SY5Y cells. SH-SY5Y cells were cultured in serum-free DMEM-F12 (1:1) in the presence of NMB (10^−8^ M or 10^−7^ M) or NGF (25 ng/mL) for 2 days. SH-SY5Y cells cultured in serum-free DMEM-F12 (1:1) were used as negative controls (NC) and those cultured in DMEM-F12 (1:1) containing 5% FBS (5% FBS) were used as positive controls. The cell viability observed in SH-SY5Y cells cultured in DMEM-F12 (1:1) containing 5% FBS was calculated as 100%, and the relative viability is shown (n = 8 per group). * and **: P<0.05 and P<0.01, respectively vs. negative control.

To examine the mechanism by which NMB protected nerves, SH-SY5Y cells, which are derived from a neuroblastoma cell line and can be induced to differentiate into neuron-like cells, were cultured in the presence of NMB, and the effect of NMB on neuron differentiation was examined by measuring the length of neurites. NMB did not significantly elongate neurites, whereas NGF significantly increased neurite length, and thereby induced differentiation of SH-SY5Y cells into neuron-like cells (Fig 7A and 7B). We also examined whether NMB promoted the survival of SH-SY5Y cells under serum deprivation. When NMB was added in serum-free medium, the survival of the cells increased significantly as compared with the negative control group, in which cells were cultured in serum-free medium during the same time course (Fig 7C). NGF also increased the survival of the cells significantly as compared with the negative control. Collectively, these results suggested that NMB stimulated the survival of SH-SY5Y cells, although it did not promote the neuron-like differentiation of these cells.

## Discussion

In this study, we demonstrated that overexpression of NMB restored erectile function in diabetic rats, as assessed by ICP measurements. The recovery of erectile function in the AdNMB-injected rats was associated with the recovery of the expression of VE-Cad, SMA, and nNOS. These results suggested that NMB recovered erectile function via prevention of the destruction of the cavernous body and promotion of the survival of nitrergic nerves in the penis. Gene transfer of molecules, including eNOS, nNOS, ion channels, calcitonin gene-related peptide, and IGF-1, restored erectile function in animal models [4–7, 9]. We have recently demonstrated that AM and Ang-1 restored erectile function via protection of the cavernous body from injury[16, 17]. To the best of our knowledge, this is the first report to demonstrate that NMB can restore erectile function.

Although it is well known that NMB has various biological activities such as stimulation of gastrointestinal/urogenital smooth muscle contraction [19–21], inhibition of thyrotropin release from the pituitary gland [22, 23], induction of satiety [24, 25], and mediation of stress and fear responses [26, 27], little is known about its activities in blood vessels and peripheral nerves. It has recently been demonstrated that NBM stimulated angiogenesis *in vivo* and *ex vivo* models [29]. Additionally, expression of the receptor for NMB was detected in VECs, and NMB was shown to be capable of stimulating angiogenesis via activation of the extracellular signal-regulated kinase/phosphatidylinositol 3-kinase(PI3K)/Akt/eNOS-dependent pathways [29]. Since the expression and activity of eNOS were restored in the penis of AdNMB-injected diabetic rats compared with that in AdGFP-injected diabetic rats, as assessed by the expression of total eNOS and phospho-eNOS, NMB might stimulate angiogenesis via PI3K/Akt/eNOS-dependent pathways and promote the regeneration of cavernous body. The recovery of VE-Cad and SMA expression also supports this hypothesis. Both Ang-1 and AM have been demonstrated to activate PI3K/Akt-dependent pathways and stimulate angiogenesis [36, 37]. Therefore, NMB appears to restore erectile function via a similar mechanism used by Ang-1 and AM, i.e., the stimulation of angiogenesis. It is also possible that NMB enhanced the effects of endogenous pro-angiogenic molecules, such as VEGF-A. NMB-induced increases in the expression of total eNOS might enhance VEGF-A-induced angiogenesis since VEGF-A stimulates angiogenesis in an eNOS-dependent manner [38]. Another possibility is that NMB might prevent the apoptotic death of VECs and/or vascular myocytes. Although possible anti-apoptotic effects of NMB on VECs and vascular myocytes have not been reported, PD168368, an inhibitor of the NMB receptor, suppressed the growth of breast cancer cells by inducing cell cycle arrest and apoptosis [39], suggesting that NMB may have anti-apoptotic activity. It is well established that Ang-1 and AM have anti-apoptotic activity in VECs and vascular myocytes [40–42]. Although the role of Ang-1 and AM anti-apoptotic activity in the restoration of erectile function also remains to be determined, it is possible that Ang-1, AM, and NMB may all restore erectile function at least in part through their anti-apoptotic activity in blood vessels.

Since the expression of nNOS was recovered when the AdNMB was injected into the penis, we hypothesized that NMB stimulated the differentiation of nerve cells and/or protected nerve cells from death. To examine these possibilities, we used SH-SY5Y cells and examined whether NMB stimulated their differentiation and survival. Although NMB did not induce differentiation of SH-SY5Y cells into neuron-like cells, it did promote their survival. Despite the fact that little is known about the activities of NMB in peripheral nerves, NMB expression has been detected in nociceptive sensory neurons and found to be capable of inducing sensitization of peripheral neurons to thermal and mechanical stimulation [43]. These findings suggested that NMB had physiological activities in peripheral nerves as well as in the central nervous system. Thus, it is possible that NMB stimulated the survival of nitrergic nerves in the penis, thereby restoring erectile function. Another possibility is that NMB protected the nitrergic nerves from hypoxia and low nutrition by stimulating angiogenesis. Future studies will be required to elucidate these points.

In conclusion, NMB restored erectile function via protection of the cavernous body from injury and promotion of the survival of nitrergic nerves. NMB may therefore represent a new candidate molecule that can be used for the treatment of ED.
